# Hepatitis C microelimination among people living with HIV in Taiwan

**DOI:** 10.1080/22221751.2022.2081620

**Published:** 2022-06-20

**Authors:** Guan-Jhou Chen, Shu-Yuan Ho, Li-Hsin Su, Sui-Yuan Chang, Szu-Min Hsieh, Wang-Huei Sheng, Wang-Da Liu, Yu-Shan Huang, Kuan-Yin Lin, Yi-Ting Chen, Yi-Ching Su, Wen-Chun Liu, Hsin-Yun Sun, Chien-Ching Hung

**Affiliations:** a Department of Internal Medicine, National Taiwan University Hospital and National Taiwan University College of Medicine, Taipei, Taiwan; b Min-Sheng General Hospital, Taoyuan, Taiwan; c Department of Laboratory Medicine, National Taiwan University Hospital and National Taiwan University College of Medicine, Taipei, Taiwan; d Department of Clinical Laboratory Sciences and Medical Biotechnology, National Taiwan University College of Medicine, Taipei, Taiwan; e Department of Medicine, National Taiwan University Cancer Center, Taipei, Taiwan; f Department of Tropical Medicine and Parasitology, National Taiwan University College of Medicine, Taipei, Taiwan

**Keywords:** Men who have sex with men, injection drug use, sexually transmitted disease, direct-acting antiviral, sustained virologic response

## Abstract

To reach the WHO target of hepatitis C virus (HCV) elimination by 2025, Taiwan started to implement free-of-charge direct-acting antiviral (DAA) treatment programme in 2017. Evaluating the progress of HCV microelimination among people living with HIV (PLWH) is a critical step to identify the barriers to HCV elimination. PLWH seeking care at a major hospital designated for HIV care in Taiwan between January 2011 and December 2021 were retrospectively included. For PLWH with HCV-seropositive or HCV seroconversion during the study period, serial HCV RNA testing was performed using archived samples to confirm the presence of HCV viremia and estimate the prevalence and incidence of HCV viremia. Overall, 4199 PLWH contributed to a total of 27,258.75 person-years of follow-up (PYFU). With the reimbursement of DAAs and improvement of access to treatments, the prevalence of HCV viremia has declined from its peak of 6.21% (95% CI, 5.39–7.12%) in 2018 to 2.09% (95% CI, 1.60–2.77%) in 2021 (decline by 66.4% [95% CI, 55.4–74.7%]); the incidence has declined from 25.94 per 1000 PYFU (95% CI, 20.44–32.47) in 2019 to 12.15% per 1000 PYFU (95% CI, 8.14–17.44) (decline by 53.2% [95% CI, 27.3–70.6%]). However, the proportion of HCV reinfections continued to increase and accounted for 82.8% of incident HCV infections in 2021. We observed significant declines of HCV viremia among PLWH with the expansion of the DAA treatment programme in Taiwan. Further improvement of the access to DAA retreatments is warranted to achieve the goal of HCV microelimination.

## Introduction

In June 2016, the World Health Organization (WHO) announced its first global target to eliminate viral hepatitis as a major public health threat by 2030, aiming to reduce the new diagnoses of viral hepatitis by 90% and hepatitis-related deaths by 65% [1]. This 2016 global strategy to reduce transmission of hepatitis C virus (HCV) included screening of donated blood products, harm reduction programmes targeting people who inject drugs (PWID), and expanding the access to direct-acting antiviral (DAA) treatments. Studies have demonstrated that DAA treatments could achieve a very high rate of sustained virologic response and reduce all-cause mortality, liver-related complications and occurrence of hepatocellular carcinoma [2–5]. Moreover, learning from the “treatment as prevention” strategy in combating HIV, the widespread use of DAAs could also reduce the level of circulating HCV in the community and hence prevent new HCV infections [6,7]. In Australia, a near 50% reduction of incident HCV infections has been achieved after scaling up the coverage of DAA treatments among incarcerated populations [8]. Studies in Egypt and Georgia also demonstrated significant reductions in HCV infections in the general population with the expansion of DAA treatments [9,10].

However, a nationwide hepatitis elimination programme could be extremely challenging and resource-consuming for many countries. Therefore, instead of pursuing population-wide HCV elimination, it is probably reasonable to start by targeting specific subpopulations who are at higher risk of acquiring HCV or developing HCV-related complications. One of the most commonly targeted subpopulations for this “microelimination” goal is people living with HIV (PLWH), as many epidemiological studies have demonstrated high rates of HCV coinfection due to the shared behavioural risk factors, such as sharing needles/syringes and diluent or unprotected sexual contacts [11]. Furthermore, PLWH with HCV coinfection has a much higher risk of progression to liver-related complications [12].

As of January 2022, several European countries and Australia have reported the progress of HCV microelimination among PLWH [13–16]. In the Swiss HIV Cohort Study, population-wide HCV screening followed by unrestricted access to DAA treatments has resulted in a 78% reduction of HCV incidence [16]. Similarly, in the UK, Australia and the Netherlands, studies focusing on PLWH who are men who have sex with men (MSM) also demonstrated a 49–71% reduction of HCV incidence after implementation of unrestricted access to early DAA treatment [13–15]. Studies in Switzerland also demonstrated a continuous effect of HCV control after the microelimination programme among PLWH [17]. In the Asia-Pacific region, there were also some HCV microelimination programmes in correctional facilities or prisons [18,19]. However, studies to examine the progress of HCV microelimination after DAA rollout in the Asia-Pacific region remain scarce, given the higher prevalence and incidence of HCV infection in this region [1].

In this 10-year cohort study, we aimed to investigate the trends of HCV viremia among PLWH in Taiwan before and after the implementation of the DAA treatment programme.

## Methods

### Study setting

In Taiwan, the National Health Insurance started to reimburse DAA treatments in January 2017, which initially covered only HCV-infected patients with moderate to severe fibrosis; patients who were reinfected with HCV or had relapses after DAA treatments were not reimbursed. In 2018, the government published Taiwan Hepatitis C Policy Guidelines, aiming to reach the WHO HCV elimination targets by 2025 [20,21]. As of 2021, restrictions on DAA treatment have been gradually lifted to increase the coverage of treatments and all patients with HCV viremia, regardless of chronicity, could have access to DAA treatment [22]. The second course of DAA treatment could not be reimbursed for those who had HCV relapses or reinfections following the first course of DAA treatment until early 2021. By the end of 2021, approximately 128,000 HCV-infected patients had already received DAA treatments, which accounted for 50% of the estimated treatment target [20].

In Taiwan, the total case number of PLWH diagnosed was 42,263 between 1984 and 2021 and the UNAIDS 95-95-95 targets achieved was 90-94-95 in 2021. HCV seroprevalence was estimated 43.4% among PLWH who initiated antiretroviral therapy between 2004 and 2007, which had decreased to 18.6% between 2012 and 2016, as compared to the 4% HCV seroprevalence in the general population [23]. Among risk groups of HIV transmission, more than 90.0% of PWID were HCV-seropositive while HCV seroprevalence was 3.5%–5.9% among MSM [23,24]. Over the past two decades, the incidence of HIV transmission among PWID had significantly decreased with the sustained implementation of the harm reduction programme since 2005, from its peak of 45.1% (1543/3422) of newly diagnosed HIV infections in 2005 to 2.0% or lower since 2011 [25].

### Study design and population

This was a single-centre, retrospective cohort study conducted at the National Taiwan University Hospital (NTUH), the largest designated hospitals for inpatient and outpatient HIV care in Taiwan. PLWH who were aged 18 years or older and sought HIV care at NTUH between 1 January 2011 and 31 December 2021 were included. PLWHs were included in this study since their first inpatient or outpatient visit with a confirmed diagnosis of HIV infection during the study period and were followed until death, loss to follow-up, transfer of care, or the end of observation (31 March 2022), whichever occurred first.

### Definition of HCV prevalence and incidence

PLWH included were first stratified according to the HCV serostatus. HCV-seronegative PLWH who remained negative for anti-HCV antibodies throughout their entire observation period were considered uninfected with HCV, and annual HCV RNA testing was not routinely performed. For HCV-seronegative PLWH, their HCV-negative follow-up duration was calculated as the interval between their first and last available anti-HCV results. HCV-seronegative PLWH who had only one test result of anti-HCV during the follow-up were therefore excluded. For those who had at least one positive result of anti-HCV antibodies, including those who were positive at the start of observation or those who had anti-HCV seroconversion during follow-up, all archived plasma samples were retrieved and tested for HCV RNA to confirm HCV viremia. An incident case of HCV viremia was defined as (1) PLWH with new HCV viremia following a previously negative result of plasma HCV RNA or (2) HCV seroconversion with the presence of HCV RNA in the plasma. The date of incident HCV viremia was arbitrarily defined as the time when the samples that tested positive for HCV RNA were collected. For those who were HCV-seropositive or seroconverted, their HCV-negative follow-up duration was calculated as the period without HCV viremia.

Throughout the study period, the annual prevalence and incidence rate of HCV viremia in each calendar year were calculated. The prevalence of HCV viremia was defined as the number of PLWH who had one or more plasma samples tested positive for HCV RNA (including incident and prevalent cases), divided by the total number of PLWH followed in the same calendar year. The annual incidence of HCV viremia was calculated by dividing the number of incident HCV infections (including reinfections) by the total HCV RNA-negative person-time observed during each calendar year. To compensate for potential time lags between HCV acquisition and sample collection, positive HCV RNA results within the first three months of each calendar year were assigned to the previous year in the estimations of incidence and prevalence.

### Laboratory investigations

According to the national HIV treatment guidelines in Taiwan, PLWH who were on stable antiretroviral therapy were followed at the designated hospitals every three months, and laboratory tests were performed every three to six months, including plasma HIV RNA load (PVL). In this study, all archived plasma samples during the follow-up were retrieved to test for anti-HCV antibodies and HCV RNA for those testing HCV-seropositive; those who did not have available plasma samples were excluded.

Anti-HCV antibodies were determined using a fourth-generation enzyme immunoassay (Dia.Pro Diagnostic Bioprobes S.r.l. Italy). The detection of HCV RNA was performed using the Roche Cobas^®^ 6800 system (AmpliPrep HCV Test, v2.0, Roche, USA), with a detection limit of 15 IU/ml. To facilitate early detection of HCV viremia and linkage to DAA treatments, PLWH who received a diagnosis of sexually transmitted infection (STI), had elevated aminotransferases, or achieved HCV clearance spontaneously or with DAAs were enrolled in another prospective study using three-stage pooled-plasma HCV RNA testing every three months to detect HCV viremia for 12 months after June 2019 [26]. In brief, plasma samples from 20 PLWH were pooled together for HCV RNA testing, and subsequent testing of mini-pools consisting of five samples would be performed if the initial test was positive; testing of individual samples would be performed for the mini-pooled samples tested positive for HCV RNA. Results from this study were also added to our data for better estimating the epidemics of HCV. The study design and utilization of stored plasma samples were approved by the Research Ethics Committee of NTUH (registration numbers: 201605103RINC and 201605128RINC).

### Statistical analysis

The demographic and clinical characteristics of all included PLWH were summarized. Non-categorical variables were compared using Student’s *t*-test or Mann–Whitney *U-*test, and categorical variables were compared using chi-square test or Fisher’s exact test. For non-categorical data with more than two subgroups, a one-way ANOVA test was used to detect differences between groups and pairwise comparisons with Bonferroni correction would be applied if post-hoc analysis was needed. The HCV incidence and prevalence, with a 95% confidence interval (CI), were calculated as previously defined. The differences in incidence and prevalence were compared between the end of study observation (2021), the beginning of DAA reimbursement (2017), and the epidemiological peak during observation. All statistical analyses were performed using STATA software v.14.0 S/E (StataCorp LP, College Station, TX). All *p* values were two-sided.

## Results

**Figure 1.**
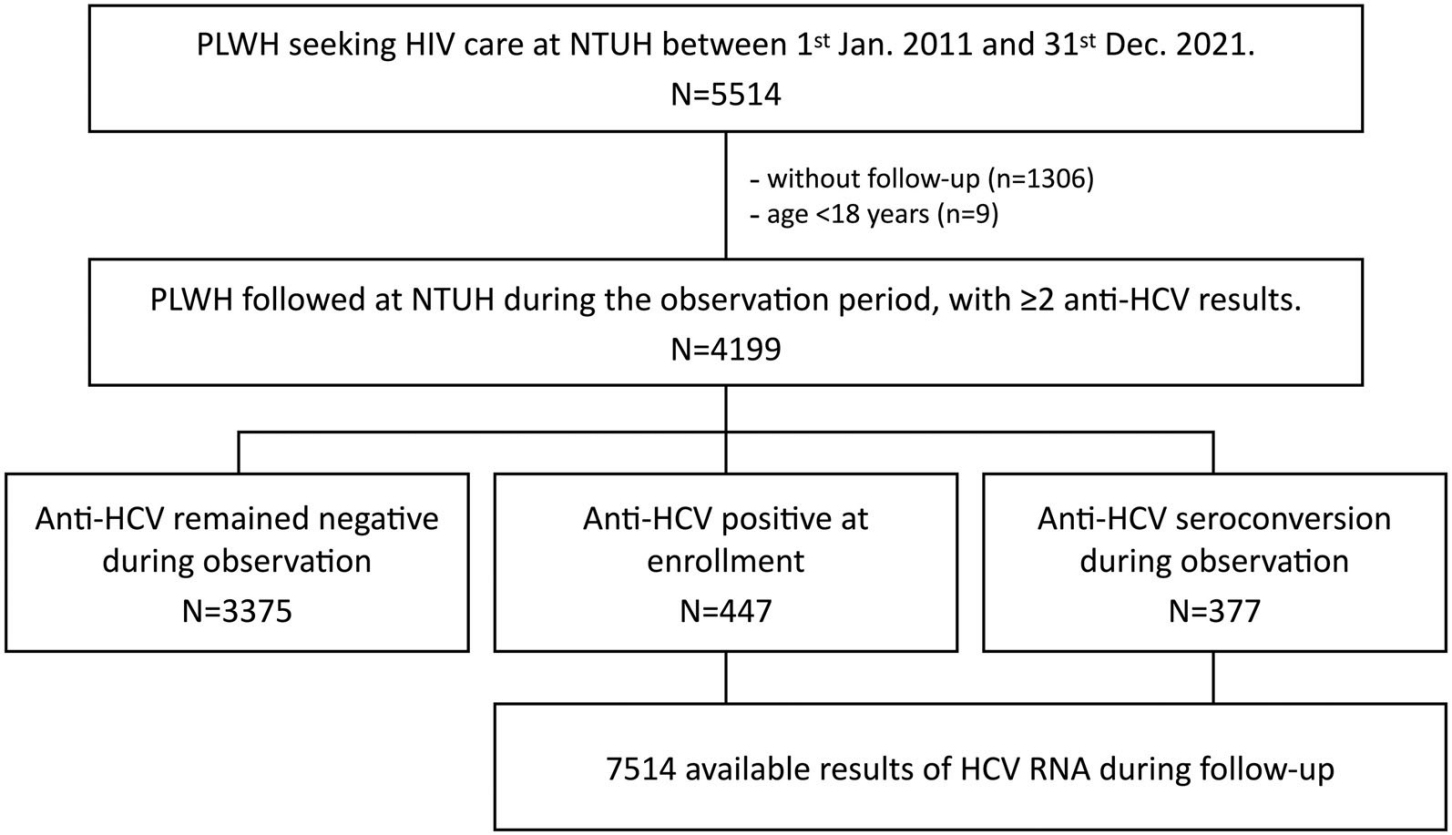
Study flow. Abbreviations: PLWH, people living with HIV; HCV, hepatitis C.

During the 10-year observation period, 5514 PLWH sought HIV care at NTUH. After excluding those who had no available samples, 4199 PLWH were included and contributed to a total of 27,258.75 person-years of follow-up (PYFU). Of all included PLWH, 3375 (80.4%) remained HCV-seronegative throughout the follow-up duration, while 447 (10.6%) were HCV-seropositive at the start of observation and 377 (9.0%) had documented HCV seroconversion during the follow-up (Figure 1). The baseline characteristics of the three groups of PLWH according to HCV serostatus are shown in Table 1.

**Table 1.** Baseline characteristics of the people living with HIV who were included in this study.

Baseline characteristics	Overall cohort	HCV seronegative	HCV seropositive	HCV seroconverted
	(*n* = 4199)	(*n* = 3375)	(*n* = 447)	(*n* = 377)
Age, mean (*SD*), years	35.4 (10.5)	35.4 (10.6)	40.0 (10.4)	30.1 (6.8)
Male sex, *n* (%)	4013 (95.6)	3244 (96.1)	392 (87.7)	377 (100)
Risk group for HIV infection				
Men who have sex with men, *n* (%)	3403 (81.0)	2829 (83.8)	213 (47.7)	361 (95.8)
Injecting drug users, *n* (%)	160 (3.8)	21 (0.6)	134 (30.0)	5 (1.3)
Anti-HCV positivity at enrolment, *n* (%)	447 (10.6)	0 (0)	447 (100)	0 (0)
HBsAg positivity at enrolment, *n* (%)	536/4100 (13.1)	420/3298 (12.7)	66/433 (15.2)	50/369 (13.5)

Abbreviations: HBsAg, hepatitis B surface antigen; HCV, hepatitis C virus; IQR, interquartile range; SD, standard deviation.

Compared with those who remained HCV-seronegative throughout the entire observation, PLWH who were HCV-seropositive at baseline were older (40.0 vs. 35.4 years, *p* < .001) and more likely to be PWID (30.0% vs. 0.6%, *p* < .001). On the other hand, those with HCV seroconversion during the follow-up were younger (30.1 vs. 35.4 years, *p* < .001) and more likely to be MSM (95.8% vs. 83.8%, *p* < .001) compared with the HCV-seronegative PLWH.

**Figure 2.**
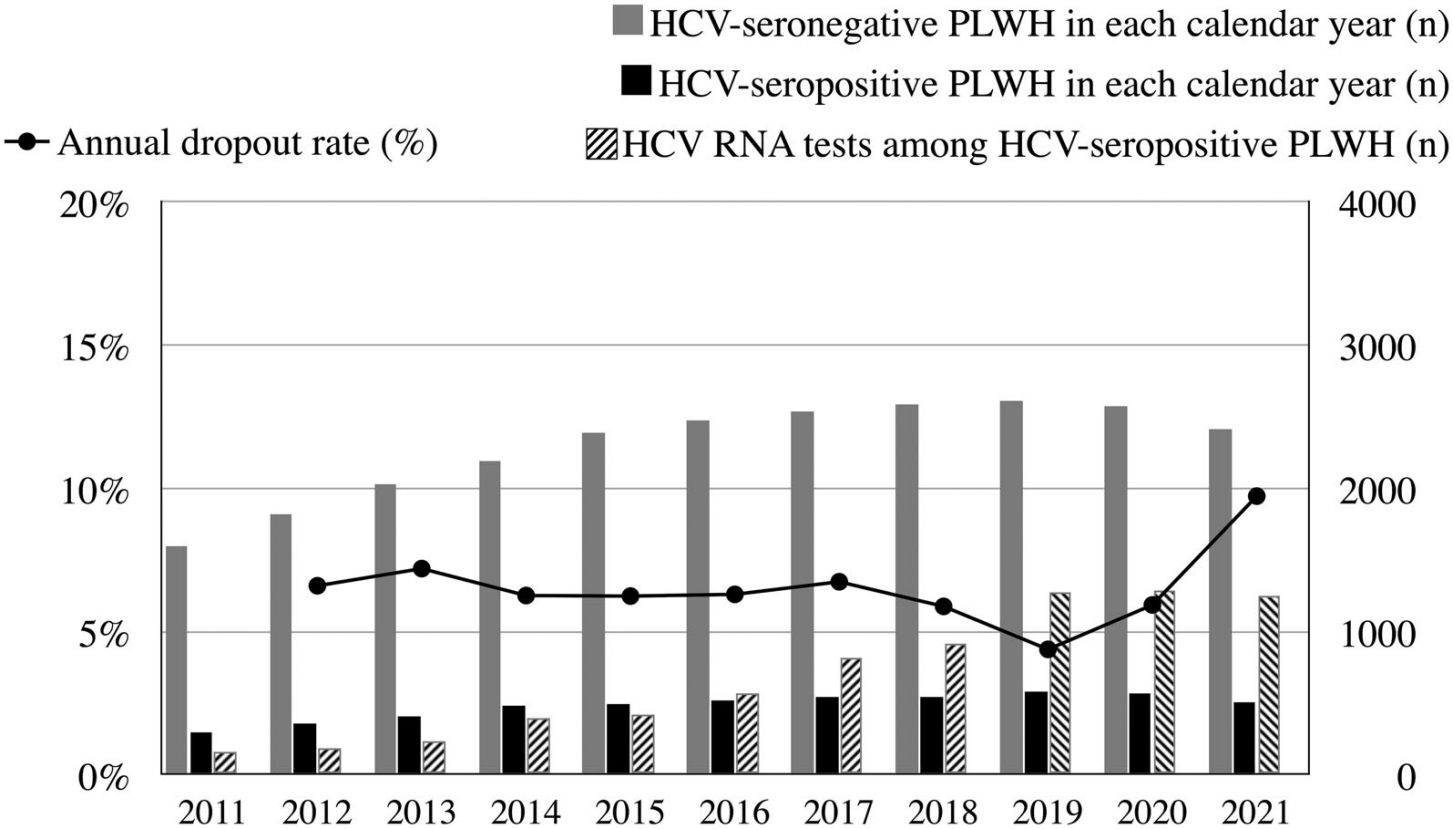
The numbers of people living with HIV (PLWH) followed, numbers of HCV RNA tests performed and annual dropout rates observed in each calendar year of follow-up. Dropouts were defined as PLWH who were included in the previous calendar year but did not have available data or samples for HCV antibody or RNA testing.

The total numbers of PLWH included in each calendar year are shown in Figure 2. The number of PLWH retained in our cohort increased gradually during the observation period and peaked in 2019 with 3195 PLWH being followed. Furthermore, the average number of HCV RNA testing for each HCV-seropositive PLWH also increased slowly with each calendar year. After 2019, on average, each HCV-seropositive PLWH had undergone more than two HCV RNA testing per year (2.19 times in 2019, 2.24 in 2020 and 2.47 in 2021).

The Coronavirus disease 2019 (COVID-19) pandemic might have hindered PLWH from seeking HIV and other medical care worldwide [27,28]. Throughout the 10 years of our study, the annual dropout rates ranged from 4.4% to 7.2% before the COVID-19 pandemic (Figure 2); however, coinciding with the peak of COVID-19 epidemic in Taiwan, the dropout rate rose to 9.7% in 2021.

### HCV incidence and prevalence

**Figure 3.**
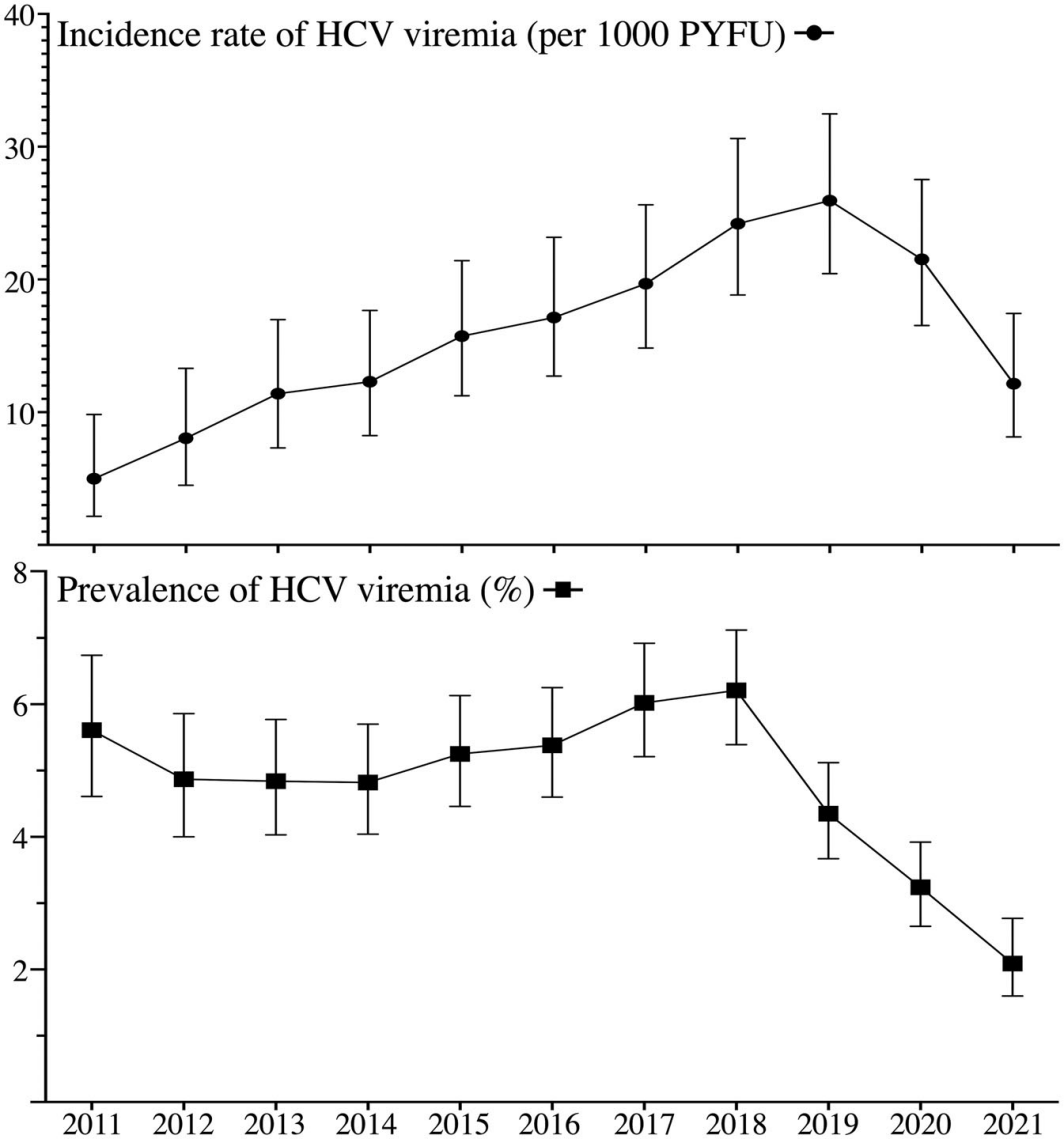
The evolution of the incidence and prevalence of HCV viremia during 2011–2021 among people living with HIV. PYFU, person-years of follow-up.

The incidence of HCV viremia was estimated at 4.99 per 1000 PYFU (95% CI, 2.61–9.83) in the beginning of this study in 2011 (Figure 3). The incidence slowly increased and peaked in 2019, with an incidence rate of 25.94 per 1000 PYFU (95% CI, 20.44–32.47). After 2019, the incidence rate of HCV viremia started to decline and reached 12.15 per 1000 PYFU (95% CI, 8.14–17.44) at the end of observation in 2021. When compared with the incidence (19.68 per 1000 PYFU, 95% CI 25.62–14.83) at the beginning of the DAA era in 2017, that of HCV viremia had decreased by 38.3% (95% CI, 1.5–62.0%) in 2021 with a reduction of 7.5 per 1000 PYFU (95% CI, 0.71–14.36). When compared with the epidemiological peak, the incidence rate of HCV viremia had declined by 53.2% (95% CI, 27.3–70.6%) with a reduction of 13.79 per 1000 PYFU (95% CI, 6.5–21.11).

The prevalence of HCV viremia was estimated at 5.61% (95% CI, 4.61–6.74%) at the beginning of observation in 2011 (Figure 3). The prevalence in our cohort started to increase after 2014 and peaked in 2018 with a prevalence of 6.21% (95% CI, 5.39–7.12%). After 2018, the prevalence of HCV viremia started to decrease before the decline of HCV incidence and reached 2.09% (95% CI, 1.60–2.77%) at the end of observation in 2021. When compared with the prevalence at the beginning of DAA reimbursement in 2017, that had decreased by 65.3% (95% CI, 53.9–73.9%) at the end of observation in 2021. When compared with the peak in 2018, the prevalence of HCV viremia had declined by 66.4% (95% CI, 55.4–74.7%) (Figure 3).

### First HCV infection versus reinfection

**Figure 4.**
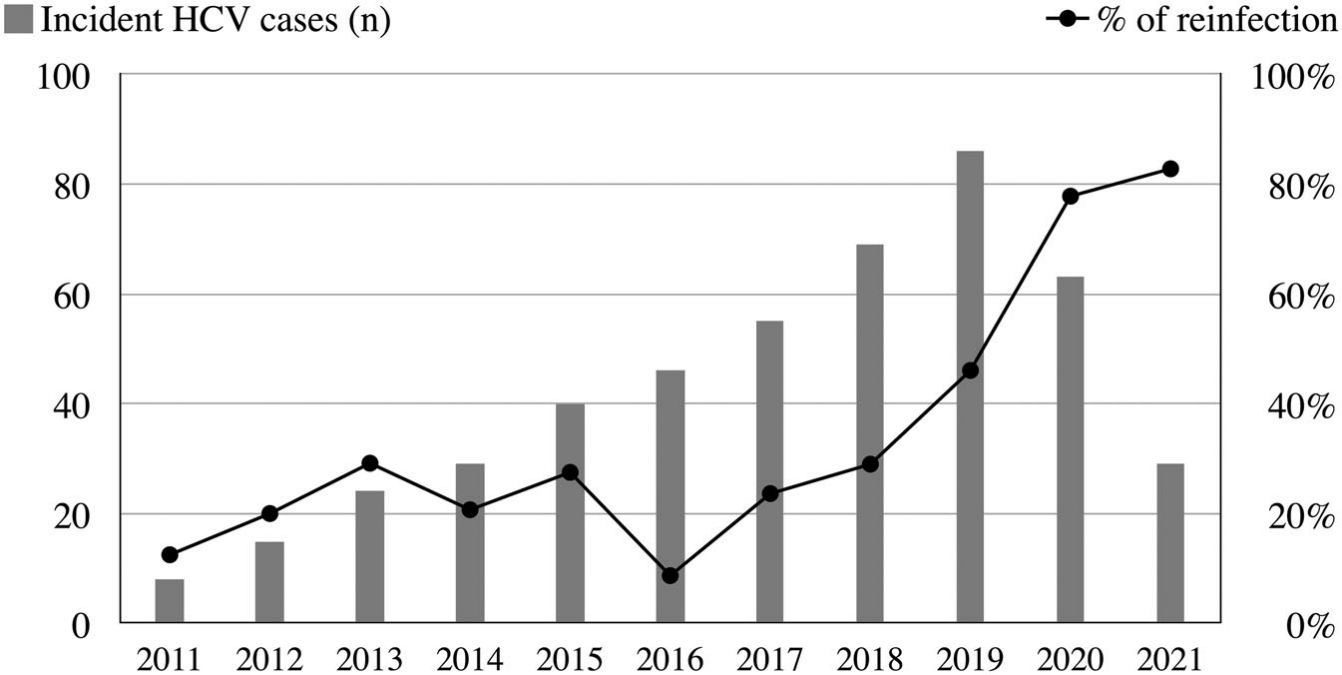
Cases of incident HCV viremia and the proportions of HCV reinfection during 2011–2021.

With each incident case of HCV viremia, we further differentiated PLWH who had the first episode of HCV infection from those who were reinfected after clearance of HCV RNA either spontaneously or by DAA treatments (Figure 4). Before the reimbursement of DAA treatments (2011–2016), around 10–30% of incident HCV infections were classified as reinfections. However, the proportion of HCV reinfection started to increase significantly after 2017. Despite the significant declines in HCV incidence and prevalence, HCV reinfections had accounted for 82.8% of incident cases of HCV viremia among our PLWH in 2021.

## Discussion

In this cohort study examining the progress of HCV microelimination among PLWH in Taiwan, we observed significant reductions in prevalence and incidence of HCV viremia after the rollout of DAA treatments in 2017. The highest prevalence of HCV viremia was observed in 2018 (6.21%; 95% CI, 5.39–7.12%) and has reduced by 66.4% (95% CI, 55.4–74.7%) at the end of observation in 2021. The incidence rate of HCV viremia has declined by 53.2% (95% CI, 27.3–70.6%) from a peak rate of 25.94 per 1000 PYFU (95% CI, 20.44–32.47) to 12.15 per 1000 PYFU (95% CI, 8.14–17.44).

To the best of our knowledge, this is the first longitudinal observational cohort study in the Asia-Pacific region to examine the progress of HCV microelimination among PLWH in the DAA era. Our findings of significant progress made towards HCV microelimination among PLWH in Taiwan are in line with the observations of a 49–78% reduction of HCV incidence in the UK, Australia, the Netherlands and Switzerland that expanding the accessibility to DAA treatments reduces the incidence of HCV infection among PLWH [13–16]. It is noteworthy to emphasize that the peak incidence rate observed in our cohort (25.94 per 1000 PYFU) was much higher than those from the European or Australian studies (ranging from 5.5–14.6 per 1000 PYFU) [13–16]. However, despite this high rate of HCV viremia in our cohort, a nationwide DAA rollout could still achieve a similar magnitude of HCV control as compared with the previous studies.

We previously have shown the rate of HCV seroconversion continued to increase from 2017 to 2018 in our PLWH cohort despite the reimbursement of DAAs in 2017 [29]. Starting in 2017, the National Health Insurance only reimbursed DAA treatment at hepatology clinics for those who had documented chronic HCV infection with moderate to severe liver fibrosis. As the DAA treatment became more accessible to PLWH after these restrictions were gradually lifted by involving HIV-treating physicians to provide DAA treatments to PLWH regardless of the chronicity of HCV viremia between 2017 and 2019 [20], our study demonstrated the prevalence of HCV viremia began to decrease after 2018 and the reduction of HCV incidence after 2019. The findings suggest that unrestricted access to DAA treatments is crucial for the success of HCV elimination to be achieved.

In our cohort, we observed a significantly increased proportion of incident HCV viremia attributable to reinfections, particularly after 2017. Despite the significant reductions in HCV incidence after 2019, 77.8% of incidents of HCV viremia in 2020 and 82.8% in 2021 were classified as reinfections. After the introduction of DAAs, HCV reinfections after viral clearance have raised significant concerns, especially among high-risk populations such as PWID or MSM. The rates of HCV reinfection vary widely in different geographic locations and subpopulations [30–38]. Studies in Taiwan had estimated the annual rate of HCV reinfection at 5.8–9.8% among PLWH after anti-HCV treatments [36–38]. Our findings suggest that, after initial reductions in HCV prevalence and incidence, the sustained success of the HCV elimination programme might eventually pivot to preventing reinfections among high-risk individuals. In addition to providing information, education and counselling on HCV infection, lifting the restrictions on access to DAAs for those with reinfections cannot be overemphasized.

Our studies had several limitations. First, the frequency of HCV RNA testing was not standardized. The inconsistency of testing might cause potential time lags between HCV acquisition and diagnosis. To compensate for this potential bias, we assessed the archived samples from their regular clinical visits. Second, we did not attempt to differentiate HCV reinfection from treatment failure in this study, which might lead to overestimating the incidence of HCV reinfection before the DAA era. However, considering the extremely high success rates of DAA treatments [39], we believed the bias was minimal in the DAA era. Finally, the COVID-19 outbreak after 2020 might have an adverse impact on access to HCV testing and treatment (Figure 2). The dropout rate increased to 9.7% in 2021, coinciding with the peak of the COVID-19 epidemic in Taiwan. While the increased dropout rate might reflect that some PLWH had transfer of care at other services, the average number of HCV RNA tests among HCV-seropositive PLWH did not decrease during 2019–2021. Therefore, we believe that our observation should still be representative despite the impact of the COVID-19 pandemic.

In conclusion, we demonstrate that, compared to the epidemiological peaks, the incidence and prevalence of HCV viremia had a 53.2% and 66.4% reduction, respectively, with the rollout of DAA treatments in Taiwan. However, addressing HCV reinfections among high-risk subpopulations should be a critical step in sustaining the success of HCV microelimination.

## Data Availability

The preliminary data of the present study have been presented as an ePoster (no. 1717) in Conference on Retroviruses and Opportunistic Infections (CROI) 2022.
